# Utility of CPR Machine Power and Change in Right Atrial Pressure for Estimating CPR Quality

**DOI:** 10.1038/s41598-019-45749-0

**Published:** 2019-06-25

**Authors:** Do-Yeon Lee, Seong-Min Kang, Seong-Wook Choi

**Affiliations:** Department of Mechanical and Biomedical Engineering, Kangwon National University, Chuncheon, Korea

**Keywords:** Biomedical engineering, Mechanical engineering

## Abstract

When a cardiac arrest occurs, it is necessary to perform cardiopulmonary resuscitation (CPR) as soon as possible. This requires maintaining the pressure depth at 5 cm at a rate of 100 cpm. For CPR machines, which are frequently used in ambulances, the return of spontaneous circulation (ROSC) is not superior to that of manual CPR, although CPR machines can maintain the compression rate and reciprocal distance of the compression plate more accurately. When the thoracic cavity is deformed due to repeated chest compressions, CPR machines must be adjusted. It is necessary to develop a method for measuring whether adequate CPR is achieved using CPR machines. CPR was performed on two pigs with a CPR machine, commencing 1 minute after the heart was stopped. Four CPR modes were used, with compression rates of 60 or 100 cpm and compression depths of 3 or 5 cm. The CPR machine was equipped with a load cell for measuring compression force, and a potentiometer for measuring compression depth. The measurement results obtained from the sensor were used to calculate the frequency components. The compression force and depth data were used to calculate the mechanical power of the CPR machine and mechanical impedance of the thoracic cavity. Changes in end-tidal carbon dioxide (ETCO2), coronary perfusion pressure (CPP), carotid blood flow (CBF), and right atrial pressure (RAP) were measured during performance of CPR; change in RAP refers to variation therein with chest compressions. Continuous CPR in both animals resulted in deformation of the chest cavity and a steady decline in impedance. The correlation between CPR power and change in RAP was 0.78, and that between compression force and CBF was 0.64. Impedance was not correlated with blood pressure or CBF. When the condition of the animal deteriorated due to cardiac arrest, the CPP decreased and ETCO2 increased. The CPR power and RAP varied according to the CPR mode rather than the condition of the animal. Measuring the CPR machine power does not require a separate procedure, such as catheter intubation, so should be suitable as an index of the quality of CPR in emergency situations.

## Introduction

To improve the quality of cardiopulmonary resuscitation (CPR), the American Heart Association (AHA) recommends that feedback be provided to individual performing the procedure, so that the chest compression rate remains at 100 cpm and the compression depth at 5 cm^1^. CPR machines are expected to improve the effectiveness of CPR, because they can maintain stable and continuous chest compressions. Thus, such machines have been used instead of medical staff and are highly reliable in narrow places, such as ambulances and helicopters. However, determining whether a CPR machine presses the chest to the appropriate depth can be difficult, due to deformation of the chest cavity and deviation due to vibration. CPR machines have not yet achieved superior performance to manual CPR, and we require a method for determining precisely how effectively these machines can perform the procedure^2^. The AHA recommends measuring end-tidal carbon dioxide (ETCO2) as a proxy for CPR quality, where the amount of carbon dioxide released through the lungs increases according to the blood flow stimulated by CPR^3–7^. However, according to individual differences in the characteristics of the lungs and circulatory system, the amount of carbon dioxide in the blood increases as the duration of the circulatory arrest increases; the amount of gas inhaled and discharged through the respiratory system also varies, making it difficult to assess whether CPR is being performed effectively^8^. Many studies have shown that measuring the coronary perfusion pressure (CPP), as derived from the diastolic pressure and right atrial pressure (RAP), as an alternative to ETCO2 can aid assessment of the effectiveness of CPR^9^. If the patient has been in cardiac arrest for a long time, or if CPR has not been adequately performed, the aortic pressure will significantly decrease due to vascular shock^10^. To determine whether a CPR machine is achieving adequate compression of the thorax, the patient’s condition should be checked, and appropriate changes in pressure within the thoracic cavity should be confirmed. The parameter most closely associated with chest compressions is change in RAP^11^. It is known that RAP can be affected by chest recoil, medications, and saline infusions; moreover, repeated chest compressions cause pressure changes in the entire thoracic cavity, which in turn affects RAP^12^. The CPP and RAP can only be measured by inserting a catheter into a blood vessel; however, measurements are difficult to obtain because in most cases CPR is performed outside of the hospital environment. The mechanical power of the CPR machine is expected to cause changes in blood pressure and blood flow within the patient’s chest. It is necessary to determine the effect of this power on biometric parameters and confirm its relationship with blood pressure and blood flow circulation in the thoracic cavity. In this study, we investigated the associations of CPR machine power with ETCO2, CPP, carotid blood flow (CBF), and change in RAP.

## Materials and Methods

### CPR machine setup and measurement of compression force and depth

**Figure 1:**
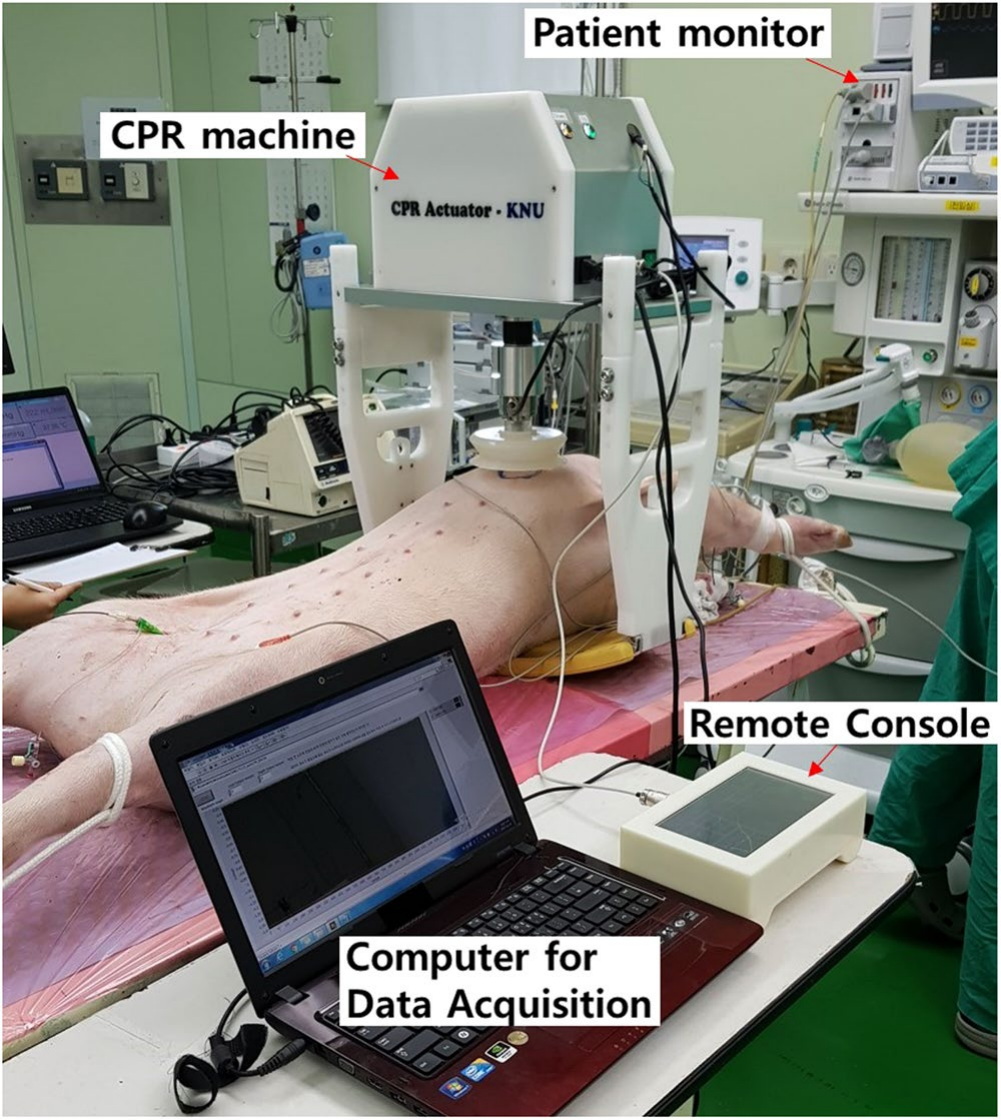
Animal experiment performed using an automatic cardiopulmonary resuscitation (CPR) machine.

As shown in Fig. 1, an electromechanical CPR machine was designed that performed chest compressions at a rate of up to100 compressions per minute (cpm), at a depth of up to 5 cm, as recommended by the AHA^3–7^. The CPR machine consists of a sensor, remote console with a graphical user interface (GUI), electromechanical actuator, compression pillar, internal battery, and patient fixation device. The compression depth is measured using an SPL0170103ST contact potentiometer (Spectra Symbol Corp., Salt Lake City, UT, USA). The compression rate and depth can be changed via the GUI, which is composed of a liquid crystal display (LCD) touchscreen. A central controller calculates the speed and direction of the motor. The mechanical impedance and power of the CPR machine, calculated based on the measured compression depth and rate, are displayed via the LCD^13^. A 250 W brushless DC (BLDC) motor was rotated in an electro-mechanical actuator, and a ball screw connected to the motor was rotated to in turn rotate the compression pillar connected to the centre of the motor. The built-in lithium-ion battery was designed to power chest compressions for up to 90 minutes under real-world conditions.

### Mechanical impedance and power measurements

**Figure 2:**
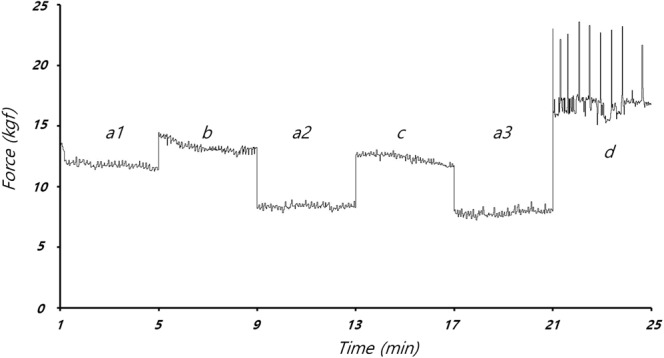
Factor of compression force at compression frequency F(k_sf_), during single-frequency analysis (SFA) under various CPR modes in animal #1.

The compression force and depth measured by the load-cell and potentiometer were converted into representative frequency components using single-frequency analysis (SFA), as detailed in the Appendix. The magnitude of the representative frequency components was measured after each compression. With the SFA method, peaks in the raw data are detected by the load cell and potentiometer to determine a single period^13^. Then, sin and cos waveforms of that period are generated, and Fourier transform performed (at the representative frequency). The SFA method can only determine the characteristics of a chest compression immediately after the end of the compression, but requires less data and fewer calculations than discrete Fourier transform (DFT). In Fig. 2, compression force was measured during the experiments using the SFA method, under six CPR modes varying by compression depth and rate (a1, b, a2, c, a3 and d). The compression force data obtained by SFA confirmed that compression force varied by mode, and with time.

**Figure 3:**
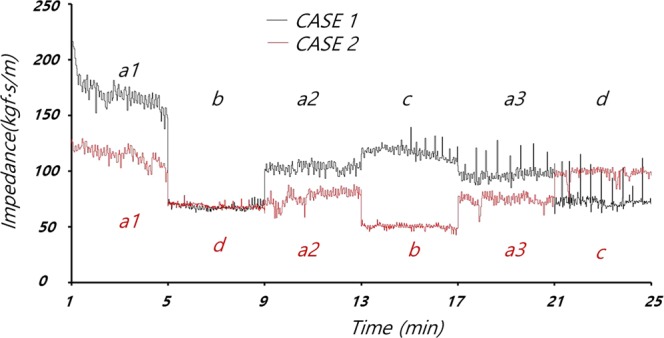
Impedance during CPR in animals #1 and #2.

**Figure 4:**
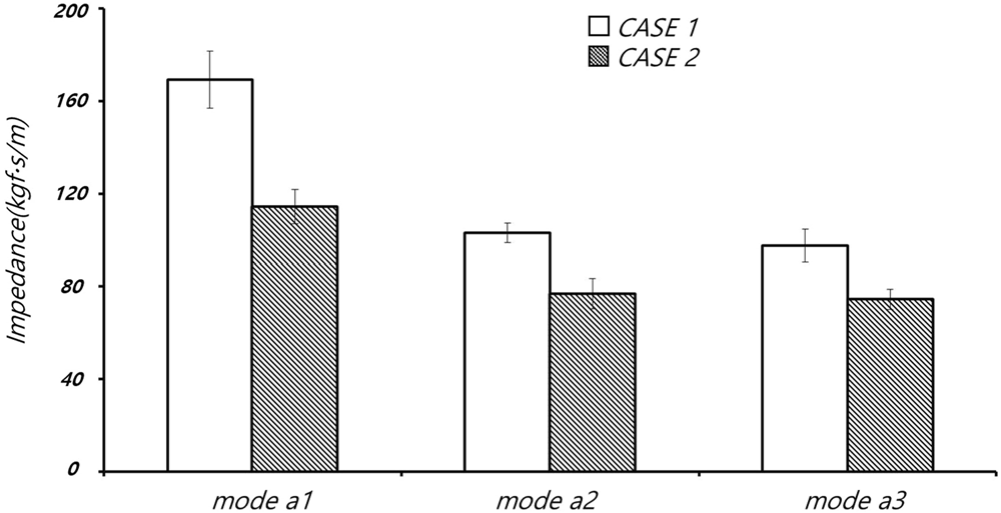
Impedance during CPR mode a.

The magnitude of the mechanical impedance of the thorax was obtained using Equation 12 (see Appendix). The mechanical impedance corresponds to changes in the mechanical characteristics of the thorax^14^. Figure 3 shows that the measured impedance varied by compression rate and depth. As shown in Fig. 4, although the compression rate and depth were the same among modes a1, a2 and a3, at 60 cpm and 3 cm, respectively, the impedance of the thorax decreased over time as the thoracic structure was deformed by repeated compressions. The magnitude of the CPR power was obtained using Equation 13 in the Appendix. The CPR power denotes the average energy transmission from the CPR machine to the animal’s chest, which can induce changes in blood pressure and blood flow in the thorax.

### Animal experiments

In induced cardiac arrest experiments, to evaluate the effect of CPR mode on power and impedance, CPR was performed on two pigs using an automatic CPR machine. The experiments were carried out in accordance with the ethical regulations of Ethics Review Committee for Animal Experimentation of Seoul National University, which approved the study protocol (approval number: IACUC 17-0107S1A2).

Prior to cardiac arrest, the animals were anaesthetised with xylazine (2 mg/kg), isoflurane (2–5%), and Zoletil (2–4 mg/kg). The pressure, electrodes and sensor of the flowmeter (FME series; Transonic Inc., Ithaca, NY, USA) used for measurement of CBF were then set up. The blood pressure readings were stable and, after cardiac arrest was induced, medications and mechanical ventilation were stopped.

ETCO2, aortic pressure, and electrocardiogram data were obtained. ETCO2 was evaluated using the side-stream measurement method with a Capstar-100 CO2 Analyzer (CWE, Ardmore, PA, USA). As an indicator of CPR quality, RAP was measured using a micro-tip pressure catheter (Millar, Houston, TX, USA) inserted via the internal jugular vein into the right atrium. The aortic blood pressure was measured with a Solar8000i monitor (GE, Boston, MA, USA). All of the measured data were acquired with a data acquisition device (PowerLab 16/34, ADInstruments Inc., USA) and stored on a personal computer. Cardiac arrest was triggered by applying a voltage of 9 V to the heart through the catheter electrode, to induce ventricular fibrillation. The CPP was given by the difference between the diastolic blood pressure (DBP) and RAP, measured during the intervals between chest compressions. The change in RAP caused by a single chest compression was obtained using the SFA method. When cardiac arrest occurred but CPR had not yet been performed, the variation in RAP was 0 mmHg.

CPR was performed from 1 minute after the induction of cardiac arrest, in respect of the latency to recognise cardiac arrest and CPR preparation time characteristic of an actual clinical situation. After the 1-minute delay, CPR modes differing in compression depth and frequency were applied. Low-quality CPR corresponded to the following CPR modes: mode a, compression depth of 3 cm and compression rate of 60 cpm; mode b, compression depth of 3 cm and compression rate of 100 cpm; and mode c, compression depth 5 cm and compression rate of 60 cpm. High-quality CPR corresponded to CPR mode d (compression depth and rate of 5 cm and 100 cpm, respectively). The CPR mode was changed using a remote console. Each CPR mode was performed for 4 minutes; ventilation was performed manually every 10 compressions with an Ambu-bag. The differences in measurement data by mode were recorded. The sampling rate was 250 Hz. When the compression rate was set to 100 cpm, the lengths of every clipped data point by using SFA were changed between 140 to 160.

In animal #1, CPR modes were applied in the order of a, b, a, c, a and d. Using SFA, the maximum compression force, F(t), was calculated as 62.04 kgf and the minimum compression force was −2.51 kgf. The factor of the compression force, F(k_sf_), varied from 7.25 to 23.58 kgf.

In the case of animal #2, the order of the CPR modes was a, d, a, b, a and c. The F(t) was 47.53 kgf, and the minimum compressive force was −1.08 kgf. The F(k_sf_) varied from 5.76 to 17.69 kgf. In both animal experiments, compression force was highest in CPR mode d, as shown in Table 1.

**Table 1:** Mean (±standard deviation) compression forces derived from single-frequency analysis of the raw data.

	Animal #1						Animal #2					
	a_1_	a_2_	a_3_	b	c	d	a_1_	a_2_	a_3_	b	c	d
Compression rate (cpm), depth (cm)	60, 3			100, 3	60, 5	100, 5	60, 3			100, 3	60, 5	100, 5
*F*(*t*) (Kgf)	17.1 ± 12.36	11.83 ± 8.97	11.3 ± 8.32	15.52 ± 11.25	13.78 ± 12.01	16.89 ± 13.37	13.53 ± 9.73	11.74 ± 7.29	11.46 ± 7.08	13.31 ± 8.40	13.75 ± 10.77	18.24 ± 13.16
*F*(*k*_*sf*_) (Kgf)	11.85 ± 0.37	8.36 ± 0.20	7.85 ± 0.26	13.28 ± 0.42	12.32 ± 0.37	16.97 ± 1.41	9.77 ± 0.41	6.72 ± 0.31	6.55 ± 0.22	9.87 ± 0.20	6.55 ± 0.31	16.82 ± 0.39

We used Pearson’s correlation coefficient to characterise the linear relationship between the CPR power and other parameters, such as the CPP, CBF, ETCO2, and RAP. Data for the correlation analysis were acquired over a 2-minute period, 1 minute after the CPR had started. The CPR mode was changed 1 minute after the data acquisition for a given mode was complete; data acquisition then restarted for the next mode after an additional 1-minute interval.

## Results

**Figure 5:**
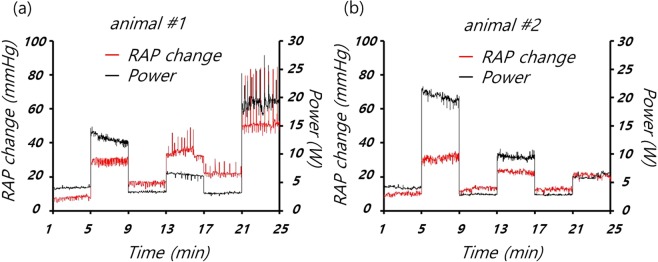
Changes in right atrial pressure (RAP) and mechanical power of the CPR machine in (**a**) animal #1 and (**b**) animal #2.

In the animal experiments, mechanical impedance decreased with repeated chest compressions, as shown in Figs 3 and 4. The impedance was weakly correlated with the CPR mode. Figure 5 shows that the mechanical power of the CPR machine was affected less by deformation of the thorax than the impedance, where this also differed among the CPR modes. Table 2 shows the data for CPR power, impedance, ETCO2, CPP, CBF and RAP by CPR mode.

**Table 2:** Mean (±standard deviation) compression forces derived from single-frequency analysis, plus mechanical impedance, CPR power and biometric parameters.

CPR mode (number of data points)	Animal #1				Animal #2			
	a (120)	b (200)	c (120)	d (200)	a (120)	b (200)	c (120)	d (200)
Compression rate, depth	60 cpm, 3 cm	100 cpm, 3 cm	60 cpm, 5 cm	100 cpm, 5 cm	60 cpm, 3 cm	100 cpm, 3 cm	60 cpm, 5 cm	100 cpm, 5 cm
*F*(*k*_*sf*_) (kgf)	11.85 ± 0.37	13.28 ± 0.42	12.32 ± 0.37	16.97 ± 1.41	9.77 ± 0.41	9.87 ± 0.20	11.05 ± 0.31	16.82 ± 0.39
Impedance (kg∙s/m)	169.35 ± 12.34	67.83 ± 2.37	117.01 ± 5.49	73.46 ± 7.97	114.25 ± 7.41	50.19 ± 1.92	98.01 ± 4.54	68.66 ± 2.05
Power (W)	4.07 ± 0.15	12.75 ± 0.65	6.37 ± 0.30	19.24 ± 1.56	4.10 ± 0.19	9.53 ± 0.37	6.11 ± 0.38	20.19 ± 0.70
CPP (mmHg)	10.80 ± 0.58	22.65 ± 1.91	18.03 ± 3.37	8.25 ± 0.73	8.79 ± 1.67	10.55 ± 1.85	13.53 ± 1.54	11.07 ± 4.26
RAP (mmHg)	7.80 ± 1.99	28.96 ± 2.49	33.98 ± 4.59	52.07 ± 7.35	12.12 ± 8.18	22.41 ± 2.66	20.81 ± 1.13	30.11 ± 4.61
ETCO2 (mmHg)	15.47 ± 2.78	22.01 ± 4.89	15.01 ± 2.19	17.01 ± 2.49	22.19 ± 4.83	34.92 ± 0.91	36.08 ± 4.51	32.27 ± 2.09
CBF (ml/min)	17.93 ± 2.55	46.34 ± 7.43	21.48 ± 6.46	59.19 ± 11.55	21.90 ± 3.31	55.34 ± 8.41	42.52 ± 15.38	53.92 ± 12.79
ABP (mmHg)	6.03 ± 0.99	16.64 ± 2.30	18.11 ± 1.44	30.54 ± 4.42	8.31 ± 1.57	14.48 ± 2.42	15.04 ± 1.64	19.06 ± 8.04

**Figure 6:**
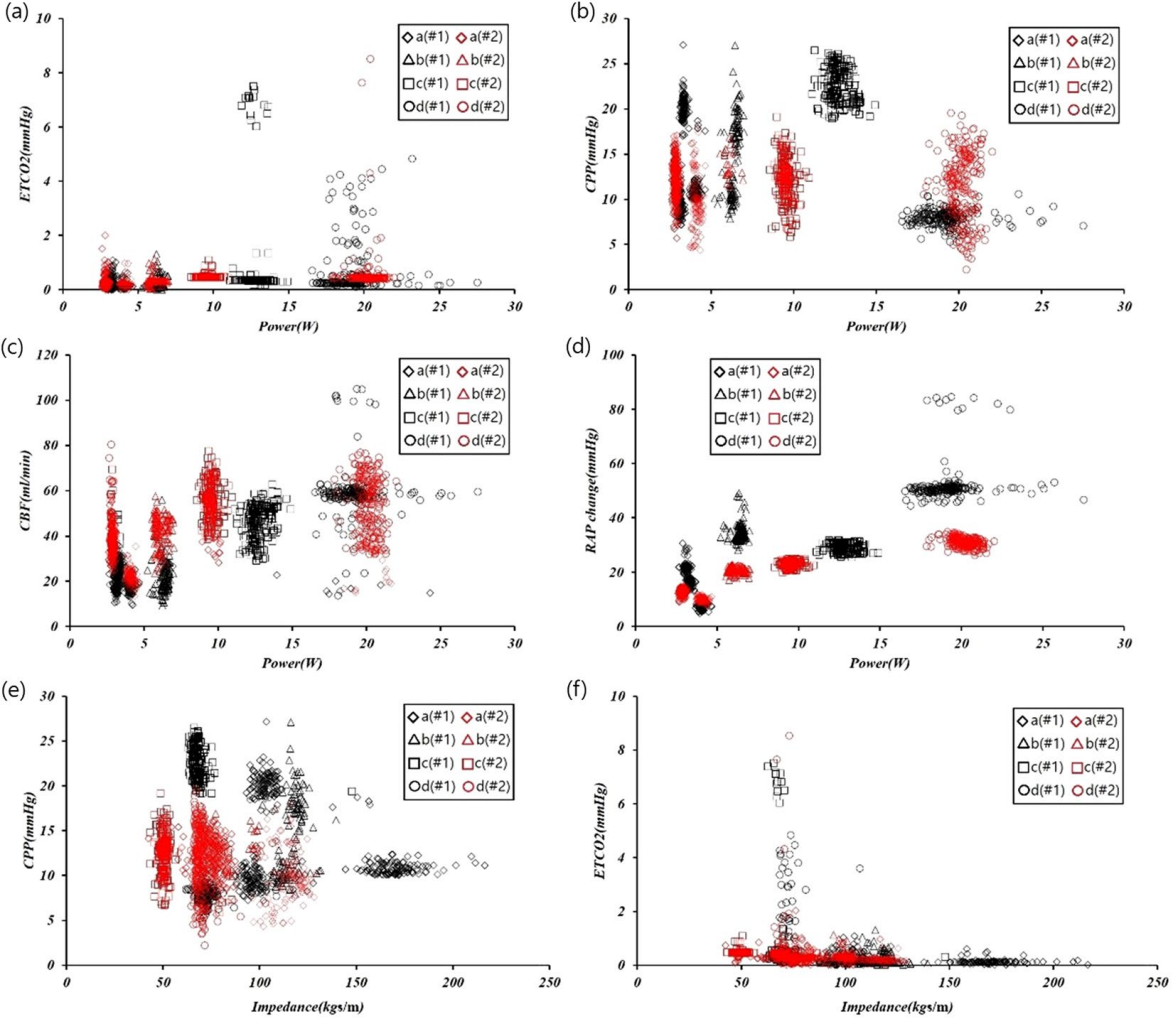
Relationships of biometric data, CPR power and mechanical impedance under the various CPR modes.

Figure 6 shows the association of the CPR power and mechanical impedance with the other biometric parameters of interest according to CPR mode. The Pearson correlations between the CPR power and ETCO2, CPR power and CPP, CPR power and CBF, CPR power and change in RAP, mechanical impedance and CPP, and mechanical impedance and ETCO2 were 0.24, 0.10, 0.64, 0.78, 0.33, and 0.19, respectively.

## Discussion and Conclusion

The compression plate of the CPR machine is reciprocated up and down from an initially fixed position, to compress the thorax to a constant depth; if the thorax becomes deformed due to repeated chest pressure, then the compression plate of the CPR may fall off the thorax resulting in ineffective chest compressions. The mechanical forces measured in CPR modes a1, a2 and a3 (see Fig. 2) decreased with additional chest compressions due to the decrease in the elastic modulus of the thoracic structure, which occurred for unknown reasons but may have involved partial fracture of the rib or ligament damage. In CPR modes a1, a2, a3 (chest compression rate and depth of 60 cpm and 3 cm, respectively; see Figs 3 and 4), the mechanical impedance decreased over time. Figure 3 shows that a significant change in impedance occurred during application of CPR mode a1. The CPR power remained constant during mode a1, such that the force applied to the chest was constant regardless of the mechanical impedance. As shown in Fig. 6d, the RAP and CPR power clearly differed among CPR modes, such that deformation of the thorax does not seem to have a major effect on the quality of the CPR.

Figure 5 shows the changes in RAP in the two animals, measured along with the CPR power; it can be seen that the CPR power varied by CPR mode, similar to RAP. The variation in RAP was caused by the chest compressions; although RAP can be affected by fluid injection and certain emergency medicines, such as epinephrine, changes in RAP during cardiac arrest were negligible in this study in the absence of CPR^9,11^. When a chest compression was performed by the CPR machine, the internal pressure of the thorax and RAP both increased. However, when the compression plate was retracted, the internal pressure of the thorax and RAP decreased immediately. Change in RAP can be used to predict the extent to which chest compression affects the internal pressure of the thorax. In this study, the Pearson correlation coefficient between CPR machine power and CBF was 0.64.

In recent years, CPP has frequently been used as a proxy of the quality of CPR. The CPP is calculated as the difference between the DBP and RAP, except during actual compressions. In this study, the Pearson correlation coefficient between the CPP and CPR power was only 0.1, where the DBP decreased continuously as the physiological condition of the animals deteriorated. Although the CPP is affected by chest compressions, it also seems to be affected by the condition of the patient.

The ETCO2 measurements obtained in this study were somewhat irregular, as expected due to the erratic nature of the air mass moving through the airway during the chest compressions. After 10 chest compressions and 1 manual respiration, performed using an Ambu-bag, the ventilation of the lungs increased temporarily, while the concentration of carbon dioxide decreased temporarily. The ETCO2 increased over time, apparently due to the continued accumulation of carbon dioxide in the vessels during low-quality CPR.

The CPP and ETCO2 are meaningful indicators of the patient’s condition, as well as of the effectiveness of CPR. The CPP and ETCO2 are useful for predicting the likelihood of recovery when CPR is performed appropriately^3–8^. However, if the CPR is of low quality, the CPP and ETCO2 decrease; in this study, it was unclear whether these parameters were affected by the CPR mode or the animal’s condition.

Figure 6 shows that the different CPR modes can be distinguished most obviously by the CPR power and changes in RAP. Both RAP and CPR power are relatively unaffected by the patient’s condition, and can be used to determine whether the compression depth or force should be increased. Also, CPP and ETCO2 provided important information regarding the appropriate CPR conditions.

In this study, we assessed how CPR mode affected CPR power and RAP over time; biometric data were also obtained for each animal. Figure 6 shows that the two animals differed not only in terms of RAP and CPR power, but also in CPP, ETCO2, and CBF, due to differences in their chest structures and condition, and the order in which the various CPR modes were applied. Results would likely vary significantly in other animal species or humans, and additional studies are required to precisely elucidate the determinants of CPR power and RAP changes. The CPR power can be obtained easily in an emergency situation, because its measurement does not require insertion of a catheter or sensors, unlike CPP and ETCO2; it can be useful for determining whether the CPP and ETCO2 are in acceptable ranges, where such parameters are useful for establishing the patient’s condition.

## Supplementary information

Appendix

## Data Availability

All data and material were obtained or produced through recognised legal procedures. They have not been published elsewhere and are not under consideration by any other journal.
