# Supplementary material for: Utility of CPR Machine Power and Change in Right Atrial Pressure for Estimating CPR Quality
Source: Sci Rep. 2019 Jun 25;9:9250. doi: 10.1038/s41598-019-45749-0 (PMC6592918; doi:10.1038/s41598-019-45749-0)
Supplement: Supplementary file 1 — Appendix [file 41598_2019_45749_MOESM1_ESM.doc]

**Utility of CPR Machine Power and Change in Right Atrial Pressure for Estimating CPR Quality**

Do-Yeon Lee^1^

E-mail: ldyeon00@kangwon.ac.kr

Seong-Min Kang^1^

E-mail: ng0213@kangwon.ac.kr

Seong-Wook Choi^1*^

E-mail: swchoe@kangwon.ac.kr

^*^Correspondence to [swchoe@kangwon.ac.kr]

^1^ Department of Mechanical and Biomedical Engineering, Kangwon National University, Chuncheon, Korea

Seong-Wook Choi^1*^

KNU Chuncheon campus, 1

Gangwondaehakgil, Chunchon-si, Gangwon-do, 24341 Republic of KOREA

Telephone: 82-33-250-6319

E-mail: swchoe@kangwon.ac.kr

**Appendix**

Single-frequency analysis: pertinent equations

When the number of data points on which to perform the frequency analysis is N_sf_, the n harmonics of the cosine and sine components can be calculated using Equations (1) and (2). The sizes of the harmonics are obtained using Equation (3). F(n) are the data obtained from the load cell.

$a_{r,n}=\frac{2}{N_{sf}}\sum_{r=1}^{N_{sf}} F(n-N_{sf}+r)\cos\left(\frac{2\pi rn}{N_{sf}}\right)$ (1)

$b_{r,n}=\frac{2}{N_{sf}}\sum_{r=1}^{N_{sf}} F(n-N_{sf}+r)\sin\left(\frac{2\pi rn}{N_{sf}}\right)$ (2)

$\left|F(k_{sf}n)\right|=\sqrt{\left(\sum_{r=1}^{N_{sf}} a_{r,n}\right)^{2}+\left(\sum_{r=1}^{N_{sf}} b_{r,n}\right)^{2}}$ (3)

In the SFA, the size of N_sf_ corresponds to the number of data points between the previous and subsequent peak, obtained using a peak detector. Equation (3) can also be expressed as Equation (4), wherein only the representative frequency, that is, n = 1 is obtained, without consideration of the other harmonic components.

$\left|F(k_{sf})\right|=\sqrt{\left(\sum_{r=1}^{N_{sf}} a_{r,1}\right)^{2}+\left(\sum_{r=1}^{N_{sf}} b_{r,1}\right)^{2}}$ (4)

The compression force on the chest is obtained using Equation (5), which is based on the DFT.

$\angle F(k_{sf})=\tan^{-1}\left(\frac{b_{r,1}}{a_{r,1}}\right)$ (5)

The magnitude and phase of the compression depth are calculated using Equations (8) and (9), respectively. D(n) are the data obtained from the potentiometer.

$a'_{r,n}=\frac{2}{N_{sf}}\sum_{r=1}^{N_{sf}} D(n-N_{sf}+r)\cos\left(\frac{2\pi rn}{N_{sf}}\right)$ (6)

$b'_{r,n}=\frac{2}{N_{sf}}\sum_{r=1}^{N_{sf}} D(n-N_{sf}+r)\sin\left(\frac{2\pi rn}{N_{sf}}\right)$ (7)

$\left|D(k_{sf})\right|=\sqrt{\left(\sum_{r=1}^{N_{sf}} a'_{r,1}\right)^{2}+\left(\sum_{r=1}^{N_{sf}} b'_{r,1}\right)^{2}}$ (8)

$\angle D(k_{sf})=\tan^{-1}\left(\frac{b'_{r,1}}{a'_{r,1}}\right)$ (9)

The magnitude and phase of the compression depth can be transformed into the magnitude and phase of the compression velocity using Equations (10) and (11), respectively, where fs is the sampling frequency.

$\left|V(k_{sf})\right|=2\pi\frac{1}{f_{s}N_{sf}}\left|D(k_{sf})\right|$ (10)

$\angle V(k_{sf})=\angle D(k_{sf})+90^{\circ}$ (11)

The magnitude of the impedance is expressed by Equation (12).

$\left|Z\right|=\left|F(k_{sf})\right|/\left|V(k_{sf})\right|$ (12)

The magnitude of the power is expressed by Equation (13).

(13)
